# Blood Compatibility of Sulfonated *Cladophora* Nanocellulose Beads

**DOI:** 10.3390/molecules23030601

**Published:** 2018-03-07

**Authors:** Igor Rocha, Jonas Lindh, Jaan Hong, Maria Strømme, Albert Mihranyan, Natalia Ferraz

**Affiliations:** 1 Nanotechnology and Functional Materials, Department of Engineering Sciences, Uppsala University, Box 534, 75121 Uppsala, Sweden; igor.rocha@angstrom.uu.se (I.R.); maria.stromme@angstrom.uu.se (M.S.); albert.mihranyan@angstrom.uu.se (A.M.); 2 CAPES Foundation, Ministry of Education of Brazil, Brasília, DF 70040-020, Brazil; 3 Department of Immunology, Genetic and Pathology, Uppsala University, Rudbeck Laboratory C5, 75185 Uppsala, Sweden; jaan.hong@igp.uu.se

**Keywords:** sulfonated beads, *Cladophora* nanocellulose, hemocompatibility, coagulation, complement system

## Abstract

Sulfonated cellulose beads were prepared by oxidation of *Cladophora* nanocellulose to 2,3-dialdehyde cellulose followed by sulfonation using bisulfite. The physicochemical properties of the sulfonated beads, i.e., high surface area, high degree of oxidation, spherical shape, and the possibility of tailoring the porosity, make them interesting candidates for the development of immunosorbent platforms, including their application in extracorporeal blood treatments. A desired property for materials used in such applications is blood compatibility; therefore in the present work, we investigate the hemocompatibility of the sulfonated cellulose beads using an in vitro whole blood model. Complement system activation (C3a and sC5b-9 levels), coagulation activation (thrombin-antithrombin (TAT) levels) and hemolysis were evaluated after whole blood contact with the sulfonated beads and the results were compared with the values obtained with the unmodified *Cladophora* nanocellulose. Results showed that neither of the cellulosic materials presented hemolytic activity. A marked decrease in TAT levels was observed after blood contact with the sulfonated beads, compared with *Cladophora* nanocellulose. However, the chemical modification did not promote an improvement in *Cladophora* nanocellulose hemocompatibility in terms of complement system activation. Even though the sulfonated beads presented a significant reduction in pro-coagulant activity compared with the unmodified material, further modification strategies need to be investigated to control the complement activation by the cellulosic materials.

## 1. Introduction

Cellulose beads have previously been described as sorption materials for a variety of applications, including, e.g., removal of metals [1] and dyes [2,3], and blood-related procedures [4,5,6,7]. A desired property for blood-contact materials is hemocompatibility. Blood-material interactions lead to activation of the blood cascades (coagulation and complement system), together with the activation of platelets and leukocytes [8]. Materials exposed to blood are identified by the recognition molecules of the cascade systems, leading to the generation of potent mediators of inflammation and coagulation. The activation of the complement system is initiated by the classical pathway, the lectin pathway, or the alternative pathway, resulting in activation of the central complement protein C3, generating the anaphylatoxin C3a and consequently inflammation. The activation pathways converge in the terminal pathway, where the membrane attack complex (C5b-9) is formed, causing the lysis of foreign cells through the formation of pores in their membranes. In the absence of a biological membrane, the C5b-9 complex binds to S-protein forming sC5b-9, which remains in the fluid phase. The blood coagulation cascade can be initiated by the intrinsic pathway (contact activation of coagulation factors) or the extrinsic pathway (triggered by tissue factor), to converge in the generation of thrombin. Thrombin cleaves the plasma protein fibrinogen into fibrin, finally promoting the formation of the clot [9].

In order to prevent the activation of the coagulation cascade, the anticoagulant heparin is usually administrated to patients during blood-related procedures. However, the long-term use of heparin may lead to side effects like thrombocytopenia, osteoporosis, and hyperkalemia [10]. Aiming to overcome the problems related with the systemic administration of heparin, the focus has been on developing non-thrombogenic materials. Several strategies have been proposed to render materials with anticoagulant properties such as surface modifications with antifouling agents, heparin coatings and heparin-like molecule grafting [11,12,13]. Researchers have investigated the incorporation of heparin-like structures in a wide range of polymer materials, demonstrating that sulfate and sulfonate groups influence the material's anticoagulant activity [14,15,16,17].

Our group has recently reported the synthesis of sulfonated cellulose beads prepared from *Cladophora* nanocellulose [18]. Nanocellulose from *Cladophora* green algae is a very versatile material that presents an entangled fibrous web structure, high specific surface area, high degree of crystallinity, and excellent mechanical and rheological properties [19,20]. These features have contributed to the development of *Cladophora* nanocellulose-based materials for biomedical applications such as virus removal filters [21,22,23], membranes for electrochemically-assisted hemodialysis [24,25,26], DNA-immobilized immunosorbents membranes [27], and drug delivery vehicles [28,29].

The sulfonated *Cladophora* nanocellulose beads present properties such as high surface area, high degree of oxidation, spherical shape, and the possibility of tailoring the porosity, making them interesting candidates for the development of immunosorbent platforms, including their use in extracorporeal blood treatments [18]. Also, the presence of sulfonate groups in the polysaccharide chain may modulate the thrombogenic properties of the *Cladophora* nanocellulose. Therefore, moving forward with the characterization of the novel sulfonated *Cladophora* nanocellulose beads, we investigate the hemocompatibility of the material in terms of activation of the coagulation cascade and of the complement system, as well as hemolytic activity after contact with whole blood.

## 2. Results and Discussion

**Figure 1:**
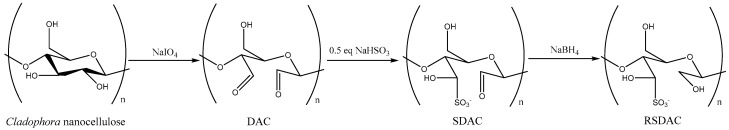
Chemical modifications of *Cladophora* nanocellulose to reduced sulfonated dialdehyde cellulose (RSDAC).

Reduced sulfonated dialdehyde cellulose (RSDAC) beads were obtained after periodate oxidation of *Cladophora* nanocellulose to 2,3-dialdehyde cellulose (DAC), with concomitant spontaneous bead formation, followed by sulfonation using bisulfite to obtain sulfonated dialdehyde cellulose (SDAC), and the reduction of the remaining aldehyde groups (Figure 1).

**Figure 2:**
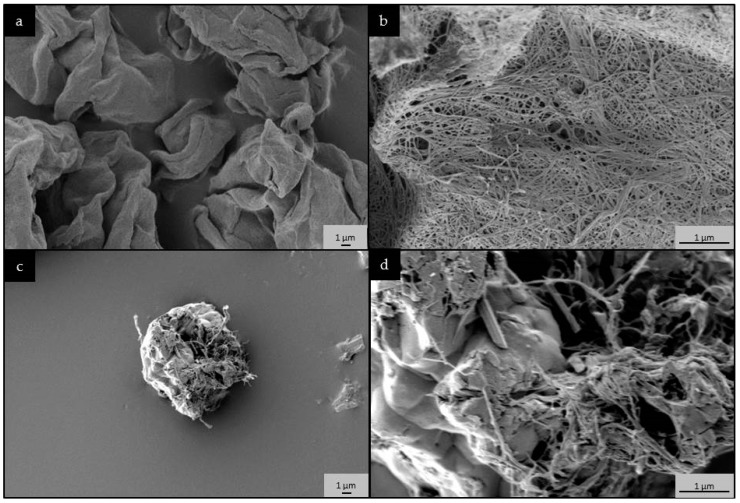
Scanning electron microscopy (SEM) images of: (**a**,**b**) *Cladophora* nanocellulose and (**c**,**d**) RSDAC materials. Observe the change in morphology from entangled fiber mesh in the unmodified *Cladophora* nanocellulose to highly porous beads in the RSDAC sample.

Scanning electron microscopy (SEM) images show the change in morphology from entangled fiber mesh in the unmodified nanocellulose to highly porous, 10–20 µm in diameter beads in the RSDAC sample (Figure 2). The size of the sulfonated beads was further confirmed by particle size distribution analysis that showed an average size of 17 µm. The sulfonation produced a highly charged material accounting for almost 500 μmol g^−1^ in comparison with the 32 μmol g^−1^ of charged groups in the starting material, which leads to a high electrophoretic mobility expressed in the ζ-potential of −25 mV (Table 1). The quantification of surface charged groups together with elemental analysis, revealed that 48% degree of sulfonation was achieved (Table 1). Both materials possess similar values of surface area, but very different pore volumes, with RSDAC showing half the value of the unmodified nanocellulose, which could be explained by the collapse of the smaller pores during the modification steps. The pore size distribution of the materials showed a shift of the mode from 13 to 40 nm when the sulfonate groups were incorporated into the cellulose material (Table 1 and Figure S1 Supplementary Information).

The hemocompatibility of the nanocellulose and the RSDAC beads was investigated by evaluating the formation of thrombin–antithrombin (TAT) complexes and the levels of complement system activation components after whole blood incubation with different amounts of the materials (concentration range 0.5–5 mg mL^−1^).

**Figure 3:**
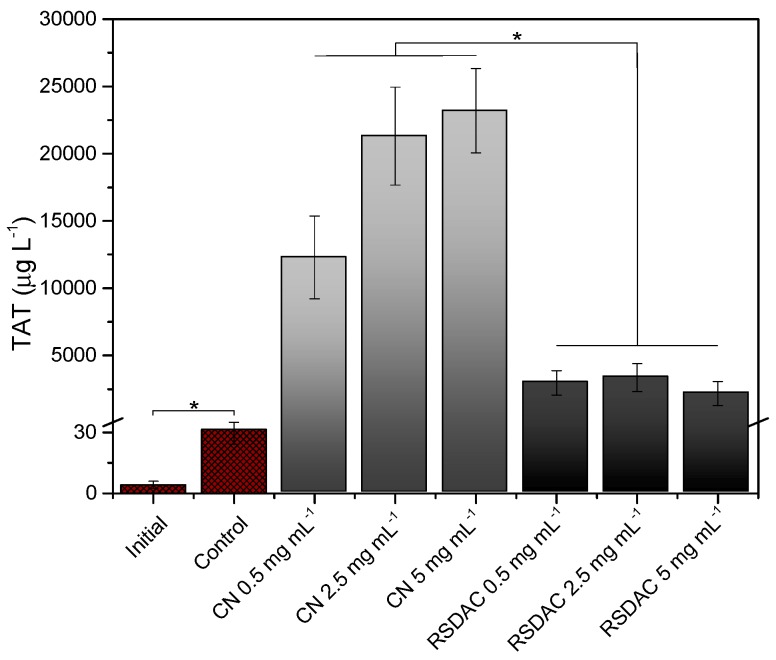
Levels of thrombin-antithrombin complexes after whole blood incubation with *Cladophora* nanocellulose (CN) and RSDAC at 37 °C for 60 min. Data represent mean ± standard error of the mean for *n* = 6. Statistically significant differences between sample groups are indicated by * (*p* < 0.05). All *Cladophora* nanocellulose and RSDAC values were significantly different from initial sample and control.

The generation of TAT complexes was selected as an indicator of coagulation activation. Thrombin is formed in the later steps of the coagulation cascade and promotes the formation of the fibrin clots from fibrinogen monomers. Antithrombin is a natural anticoagulant that binds to thrombin to control the coagulation process; thus the levels of TAT correlate with thrombin generation and therefore with pro-coagulant activity [8]. Results indicated that *Cladophora* nanocellulose was highly thrombogenic as reflected by the elevated levels of TAT (Figure 3). However, such levels decreased more than four-fold when whole blood was incubated with the RSDAC beads, indicating that a significant reduction in pro-coagulant activity was achieved by forming sulfonated cellulose beads. The retention control showed that TAT complexes were significantly retained in the nanocellulose sample (38% retention, see Table S1, Supplementary Information), while no significant differences were observed between TAT values before and after incubation with RSDAC. Therefore, the observed levels of TAT obtained with RDSAC are not a consequence of TAT retention by the sulfonated beads.

Incorporation of sulfonate groups to render materials with anticoagulant properties has been investigated by several authors. Although the type of materials (e.g., synthetic polymers, polysaccharides) varies between the studies, the reports indicate the importance of the presence of sulfate and sulfonate groups (alone or in combination with hydroxyl and amine groups) for anticoagulant activity [12,30,31,32]. The mechanism proposed for such activity also varies between the different studies, e.g., heparin-like activity [33], binding of coagulation factors [12], and interaction with fibrinogen and fibrin [34].

In the present work, the chemical modifications of the nanocellulose not only introduced sulfate groups, but also changes in the material’s morphology, pore volume, and pore size took place (Figure 2, Table 1). The complexity of the blood-material interactions is widely recognized. However, it is generally accepted that protein adsorption is a critical event during the initial response to material-blood contact and it is the adsorbed protein layer, rather than the material´s surface itself that influences blood activation [35,36]. Nevertheless, material properties like surface chemistry and functional groups, surface roughness and topography will influence the type, amount, orientation, and conformation of the adsorbed proteins, and in turn the biological response [37,38]. In the present case, it can be hypothesized that the distinct surface chemistry, morphology and nanoporosity of the RSDAC material compared with the nanocellulose could promote distinct patterns of protein adsorption in terms of conformation and activity of the coagulation factors, which in turn affects the pro-coagulant properties, as reflected in the TAT levels. The influence of nanopore size in the pro-coagulant activity of biomaterials has been previously described [39]. Moreover, the effect of material surface properties on adsorbed fibrinogen conformation and the subsequent effect on thrombin access to its substrate has also been reported [40]. It should also be considered that the presence of sulfate groups resulted in a marked increase in the material surface charge, a fact that alone has been described to influence blood coagulation via the activation of the intrinsic pathway [36]. Further studies are needed to elucidate if other surface groups, (e.g., phosphates) giving rise to a similar change in surface charge could also reduce the pro-coagulant activity of the unmodified nanocellulose.

In order to investigate complement system activation by *Cladophora* nanocellulose and RSDAC, the formation of C3a and sC5b-9 was evaluated after whole blood incubation with the materials.

**Figure 4:**
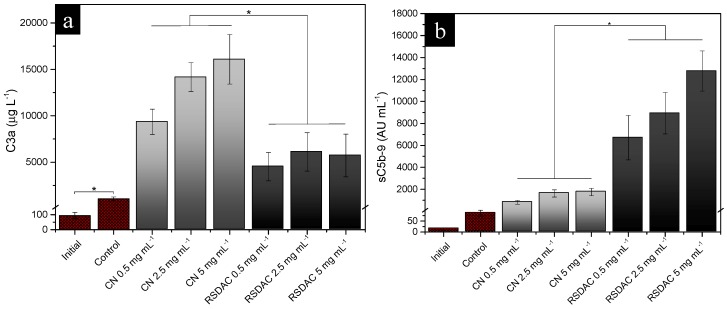
Levels of: (**a**) C3a; (**b**) sC5b-9 complexes after whole blood incubation with *Cladophora* nanocellulose (CN) and RSDAC at 37 °C for 60 min. Data represent mean ± standard error of the mean for *n* = 8. Statistically significant differences between sample groups are indicated by * (*p* < 0.05). All *Cladophora* nanocellulose and RSDAC values were significantly different from the initial sample and control.

Measurement of C3a plasma levels is usually chosen to evaluate total complement activation [41]. C5a, a potent anaphylatoxin generated in the terminal activation pathway, rapidly binds to its receptor, thus limiting its use to evaluate terminal complement activation. However, the evaluation of sC5b-9 levels is a good way of indirectly assess C5a and therefore terminal complement activation [42]. High levels of C3a were detected for the unmodified nanocellulose, while a marked reduction in such levels was observed with the RSDAC material (Figure 4a). However, the opposite trend was found with sC5b-9, i.e., higher levels were detected after blood incubation with RSDAC compared with the unmodified material (Figure 4b).

It could be speculated that the observed difference in C3a levels between *Cladophora* nanocellulose and RSDAC may be a consequence of a higher retention of the small, positively charged C3a molecule to RSDAC compared to the nanocellulose. Even though this difference could not be seen in the retention control, where C3a was readily retained by both materials (Table S1, Supplementary Information), during the whole blood experiment, the activation of the coagulation cascade might have influenced the retention of C3a as follows: a strong activation of the coagulation system takes place on the nanocellulose, with coagulation proteins, platelets and fibrin clot occupying spaces on the material and thus limiting the available space for C3a binding. On the other hand, RSDAC beads promote lower coagulation activation compared with the nanocellulose; thus a lower coverage of the material with coagulation proteins and platelets is expected, giving room for C3a binding and entrapment in the large pores of the highly negatively charged RSDAC material.

The interactions of sC5b-9 with the cellulosic materials are less affected by the differences in available binding sites and porous structures between the materials due to the large size of the complex. The retention control gave an indication that even though certain retention of sC5b-9 could be expected; there was no difference between the materials (Table S1, Supplementary Information). Thus, the observed sC5b-9 levels (Figure 4b) might be underestimated, but the difference between the materials most probably adequately represents an increase in the levels of the soluble terminal complement complex after contact with RSDAC compared with the unmodified nanocellulose.

Cellulose membranes have been classified as strong complement activators [43], a property proposed to be related with the presence of OH groups and the subsequent activation of the alternative pathway [8,44]. Thus, it is not surprising the high level of complement activation observed with the unmodified cellulosic material. On the other hand, non-activating surfaces in terms of complement system have been characterized by the presence of carboxyl, sulfate, sialic acids or bound heparin and are thought to promote high-affinity association between bound activated component C3b and the inhibitor Factor H [8]. Heparin coating of biomaterial surfaces is an example of the different strategies that have been explored to modulate material-induced complement activation [45,46,47]. In such studies, it was found that a higher concentration of bound heparin is needed to inhibit complement activation compared with the inhibition of the coagulation cascade [45]. The substitution of OH groups in cellulosic materials with different chemical groups has also been investigated in order to control the complement system response, showing that both the chemical group and the degree of chemical modification influence the activation of the complement system [48]. The degree of chemical modification could be one reason why even with the introduction of sulfate groups, a reduction in complement system could not be achieved. Future studies could address the effect of different degree of sulfonation on the complement system activation properties of *Cladophora* nanocellulose. Another reason behind the observed results could be related to the mechanism of complement system activation proposed by others [49], where an activating material is defined by its capacity to bind factor B (involved in the activation of the alternative pathway) rather than factor H (inhibitor). The high surface area of RSDAC beads could promote the adsorption of factor B, preventing an eventual positive effect of the presence of sulfate groups in inhibiting complement system activation.

It is recognized that even though progress has been made in obtaining non-thrombogenic biomaterials, successful material modifications to obtain non-complement activating biomaterials still remain a challenge for the biomaterial scientific community [9].

**Figure 5:**
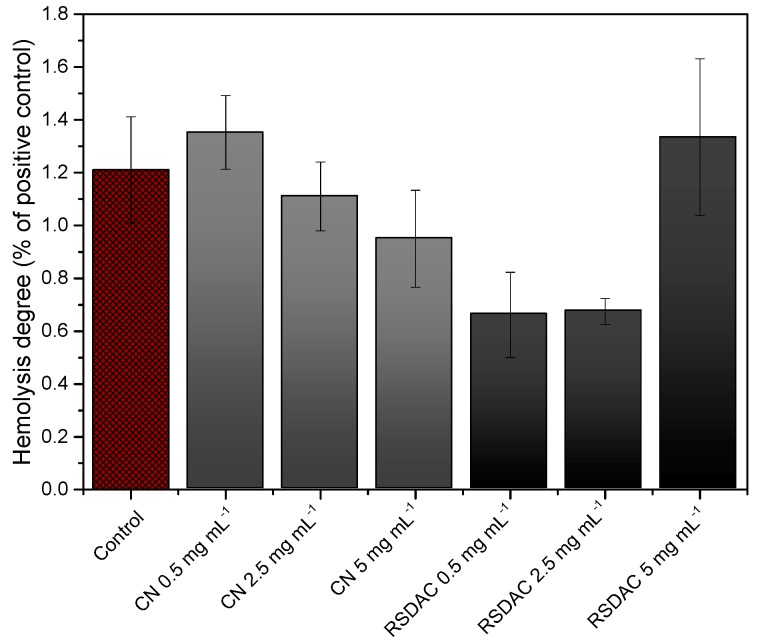
Hemolytic activity of *Cladophora* nanocellulose (CN) and RSDAC after 60 min incubation with whole blood. Results are expressed as percentage of the positive control (1% (*v/v*) Triton-X treated blood). Data represent mean ± standard error of the mean for *n* = 6.

Finally, in order to observe if the materials affect the integrity of red blood cells, the hemolytic activity was evaluated by measuring the optical density at 540 nm of plasma samples obtained after whole blood incubation with the cellulosic materials for 60 min at 37 °C. All samples showed very low values of hemolytic activity, comparable with the value obtained with the negative control (Figure 5). This is most probably due to the electrostatic repulsion between the materials and the negatively charged red blood cells, which prevent cell-material interactions.

## 3. Materials and Methods

### 3.1. Chemicals

Nanocellulose from *Cladophora* green algae was provided by FMC Biopolymer (Philadelphia, PA, USA). Sodium metaperiodate, sodium bisulfite solution, sodium borohydride, and other chemicals were of analytical or reagent grade and were used as received.

### 3.2. Preparation of Sulfonated Cladophora Nanocellulose Beads

*Cladophora* nanocellulose was converted into DAC beads following the procedure developed by Lindh et al. [50], where extensive periodate oxidation of the nanocellulose leads to spontaneous bead formation, followed by sulfonation and reduction as previously described [18]. Briefly, DAC beads were first prepared by a one-pot periodate oxidation of the nanocellulose material in an aqueous acetate buffer for 240 h at room temperature with constant magnetic stirring, converting all 2,3-hydroxyl groups to aldehydes. The never-dried DAC beads were extensively washed with water and ethanol, and further sulfonated with 0.5 equivalents of sodium bisulfite in water using the same conditions for 72 h, and named SDAC. Finally, the remaining aldehyde groups in the SDAC beads were reduced to hydroxyl groups by treating the material with sodium borohydride in the same conditions for 24 h, obtaining the RSDAC beads. In order to dry the material for the blood compatibility studies, the RSDAC beads were washed with ethanol, centrifuged, and left to air-dry for 24 h in a fume hood.

### 3.3. Materials Characterization

#### 3.3.1. Specific Surface Area (SSA) and Pore Size Distribution Analysis

The nitrogen adsorption−desorption experiments were performed at 77 K using an ASAP 2020 gas sorption instrument (Micromeritics, Norcorss, GA, USA). The samples were degassed at high vacuum (1 × 10^−4^ Pa) at 70 °C for 24 h prior to analysis. The SSA was calculated using the Brunauer-Emmett-Teller (BET) method [51] on the adsorption branch of the isotherm at P/P0 between 0.05 and 0.3. The pore size distribution was calculated using the Barrett-Joyner-Halenda (BJH) method [52] based on the desorption branch of the isotherm.

#### 3.3.2. Particle Size Measurements

Samples were dispersed in water, sonicated, and subsequently analyzed with laser diffraction using a Mastersizer 3000 instrument (Malvern Instruments, Worcestershire, UK).

#### 3.3.3. Conductometric Titration

This technique was used in order to evaluate the amount of sulfonated groups. The titrations were performed using a T70 titrator (Mettler Toledo, Greifensee, Switzerland) in 0.01 M dispersions (pH 2.8) previously sonicated and purged with nitrogen gas. The titrant was a 0.05 M NaOH solution [53].

#### 3.3.4. ζ-Potential Measurements

The electrophoretic mobility of the samples was measured using 0.001% (*w*/*w*) dispersions of the materials in NaCl_(aq.)_ (10 mM) at pH 6.5, previously ultrasonicated using a universal dip cell. The measurements were done in a ZetaSizer Nano instrument equipped with a ZetaSizer Properties Software (Malvern Instruments, Worcestershire, UK).

#### 3.3.5. Scanning Electron Microscopy (SEM)

SEM images were recorded with a Leo 1550 SEM instrument (Zeiss, Oberkochen, Germany). Samples were mounted on aluminum stubs using a double-sided adhesive carbon tape and sputtered with Au/Pd with a plasma current of 30 mA for 30 s. The thickness of Au/Pd layer was approximately 10 nm.

### 3.4. Blood Compatibility Studies

#### 3.4.1. Heparinization

Blood collecting tubes, pipette tips, loop tubings, and stainless steel connectors were heparinized to avoid unwanted blood activation. Heparinization was done according to the Corline method (Corline Biomedical AB, Uppsala, Sweden) which consists of a layer-by-layer assembly technique alternating incubations with a polymeric amine and a macromolecular heparin conjugate, to finally obtain a double-coated heparin layer.

#### 3.4.2. Blood Sampling

Fresh blood from healthy volunteers was collected in heparinized tubes containing 1 IU mL^−1^ of soluble heparin (Leo Pharma A/S, Ballerup, Denmark). The collected blood was used immediately after sampling. An aliquot of 2.0 mL of blood was collected in an Eppendorf tube containing 4 mM of ethylenediaminetetraacetic acid (EDTA) to be used as reference point (hence denoted as initial). Ethical approval for the blood experiments was obtained from the regional ethics committee (Regionala Etikprövningsnämnden Uppsala. 2008-11-06, reference number 2008/264). Informed consent from the blood donors was obtained for the experiments.

#### 3.4.3. Loop Model

**Figure 6:**
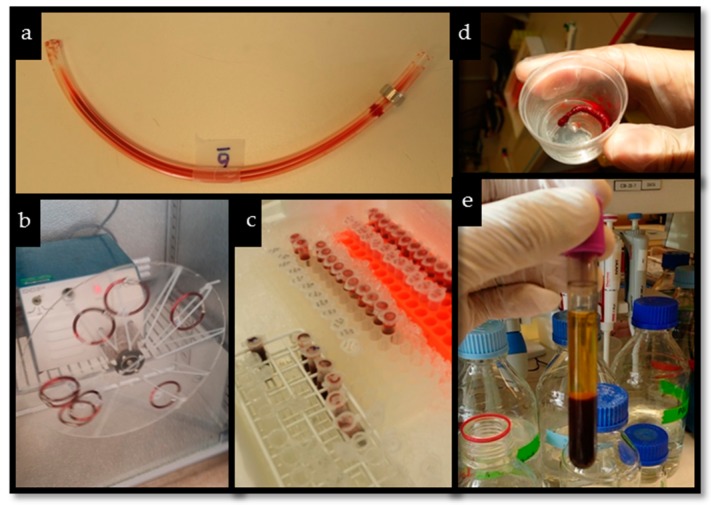
(**a**) Heparin-coated loop with blood, (**b**) closed loops with blood and material during incubation, (**c**) collected blood after incubation, (**d**) material taken from the loop after incubation with blood, (**e**) plasma-blood biphasic tube after centrifugation.

The experiments were based on the model described by Ekstrand-Hammarström et al. [54]. Heparin-coated loops with an internal diameter of 4 mm and a length of 20 cm were used for each experiment. *Cladophora* nanocellulose and RSDAC materials were added to the loops to give final concentrations of 0.5 mg mL^−1^, 2.5 mg mL^−1^ and 5.0 mg mL^−1^. In each loop 100 µL of milliQ water was added in order to soak the cellulosic materials prior to the addition of 2.0 mL of freshly drawn blood. Negative controls were included using loops filled with blood but without the cellulose materials. The loops were then closed using connectors of stainless steel and rotated vertically for 60 min in a 37 °C incubator. After each experiment, the blood was carefully collected from the loops and mixed with EDTA at a final concentration of 4 mM. The EDTA-treated blood was then centrifuged at 4500× *g* for 10 min at 4 °C. The plasma samples were collected and stored at −70 °C for further analysis. The experiments were performed with eight different blood donors and all samples were run in duplicate. Pictures of different stages of the method are shown in Figure 6.

#### 3.4.4. Enzyme-Linked Immunosorbent Assays (ELISAs) for Coagulation and Complement System Activation Markers

For all ELISAs, phosphate buffer saline (PBS) containing 1% (*w*/*v*) bovine serum albumin, 0.1% (*v*/*v*) Tween 20 and 10 mM EDTA was used as the dilution buffer, the washing buffer was PBS containing 0.1% (*v*/*v*) Tween 20; and 3,3′,5,5′-tetramethylbenzidine was used as the color substrate. Absorbance measurements at 450 nm were recorded using a Sunrise microplate reader (Tecan Group Ltd., Männedorf, Switzerland).

Control experiments were performed in order to investigate if the activation markers were retained by the materials. The cellulosic materials were incubated with standard solutions of the activation markers in the same conditions as in the loop experiments and thereafter the standard solutions were analyzed by ELISA (Supplementary Information).

##### TAT Complexes

Microtiter plate wells were coated with anti-human thrombin antibody (Enzyme Research Laboratories Inc., South Bend, IN, USA), prior to the addition of the plasma samples. Horseradish peroxidase (HRP)-conjugated anti-human antithrombin antibody (Enzyme Research Laboratories Inc., South Bend, IN, USA) was used for detection. Pooled human serum diluted in EDTA-plasma was used as a standard. Values are expressed as µg L^−1^.

##### C3a

Plasma samples were incubated in wells coated with the capture monoclonal antibody 4SD17.3 (in house). C3a was detected with biotinylated anti-C3a antibody followed by HRP-conjugated streptavidin (GE Healthcare, Uppsala, Sweden). Zymosan activated serum, calibrated against a solution of purified C3a, served as standard. The values are given in µg L^−1^.

##### sC5b-9

Plasma samples were added to microtiter plate wells coated with anti-neoC9 monoclonal antibody aE11 (Diatec Monoclonals AS, Oslo, Norway). sC5b-9 was detected by biotinylated polyclonal anti-C5 antibody (Nordic BioSite AB, Täby, Sweden) followed by HRP-conjugated streptavidin (GE Healthcare, Uppsala, Sweden). Zymosan-activated serum served as a standard. The values are presented as arbitrary units per mL (AU mL^−1^).

#### 3.4.5. Hemolysis

The hemolytic activity of each material was measured in the plasma samples by reading the optical density at 540 nm as described by Hadnagy et al. [55]. Blood sample treated with 1% (*v*/*v*) Triton-X was the positive control considered to give 100% hemolysis. The hemolytic activity of the cellulosic materials was expressed as percentage of the positive control.

### 3.5. Statistical Analysis

All results are expressed as mean values ± standard error of the mean. The software R Studio v. 0.99.489 was used to perform the statistical analysis with one way ANOVA (LSD and Tukey *post hoc* test). Normality assumptions were checked using quantile-quantile plots and log transformation of data was performed to correct for non-normality prior to the analyses. *p* values < 0.05 were considered to be statistically significant.

## 4. Conclusions

Sulfonated cellulose beads prepared from *Cladophora* nanocellulose presented a significant reduction in pro-coagulant activity compared with the unmodified material. Such improvement in pro-coagulant activity cannot only be attributed to the presence of sulfate groups, but also to the distinct physiochemical properties of the modified cellulose compared with the pristine material. Thus, to move forward with the development of non-thrombogenic cellulose beads future investigations should address the role of the physicochemical properties of the sulfonated beads on the material´s blood compatibility. The strategy of producing cellulose beads and their subsequent sulfonation did not succeed in controlling the activation of the complement system. Other modification strategies need to be explored to improve the hemocompatibility of the cellulosic materials in terms of complement system activation in order to pursue the use of sulfonated cellulose beads in blood-contact related applications.

## Figures and Tables

**Table 1:** Physicochemical properties of *Cladophora* nanocellulose and RSDAC beads. Adapted from Rocha et al. [18].

Physicochemical Properties	*Cladophora* Nanocellulose	RSDAC
Surface charged groups (μmol g^−1^)	32 ± 2 ^a^	486 ± 5 ^b^
ζ-potential at pH 6.5 (mV)	−8 ± 3	−25 ± 3
Specific surface area (m^2^ g^−1^)	96	90
Total pore volume (cm^3^ g^−1^)	0.44	0.20
Pore size distribution mode (nm)	13	40
Beads size (µm)	-	17 ± 5

^a^ carboxyl groups, ^b^ sulfonate groups. Represents 48% of degree of sulfonation.

## Supplementary Materials

The following are available online, Figure S1: Pore size distribution of *Cladophora* nanocellulose and RSDAC beads, Table S1: Levels of TAT, C3a and sC5b-9 after standard solution incubations with the studied materials.
