# Supplementary material for: Blood Compatibility of Sulfonated Cladophora Nanocellulose Beads
Source: Molecules. 2018 Mar 7;23(3):601. doi: 10.3390/molecules23030601 (PMC6017088; doi:10.3390/molecules23030601)
Supplement: Supplementary file 1 [file molecules-23-00601-s001.pdf]

Blood compatibility of sulfonated *Cladophora* nanocellulose beads

Igor Rocha,[a,b] *Jonas Lindh,*[a,*] *Jaan Hong,*[c] *Maria Strømme,*[a] *Albert Mihranyan,*[a] *and Natalia Ferraz*[a,*]

[a]Nanotechnology and Functional Materials, Department of Engineering Sciences, Uppsala University, Box 534, 75121, Uppsala, Sweden.

[b]CAPES Foundation, Ministry of Education of Brazil, Brasília - DF 70040-020, Brazil

[c]Department of Immunology, Genetic and Pathology, Uppsala University, Rudbeck Laboratory C5, 75185, Uppsala, Sweden.

SUPPLEMENTARY INFORMATION

Material characterization: Pore size distribution

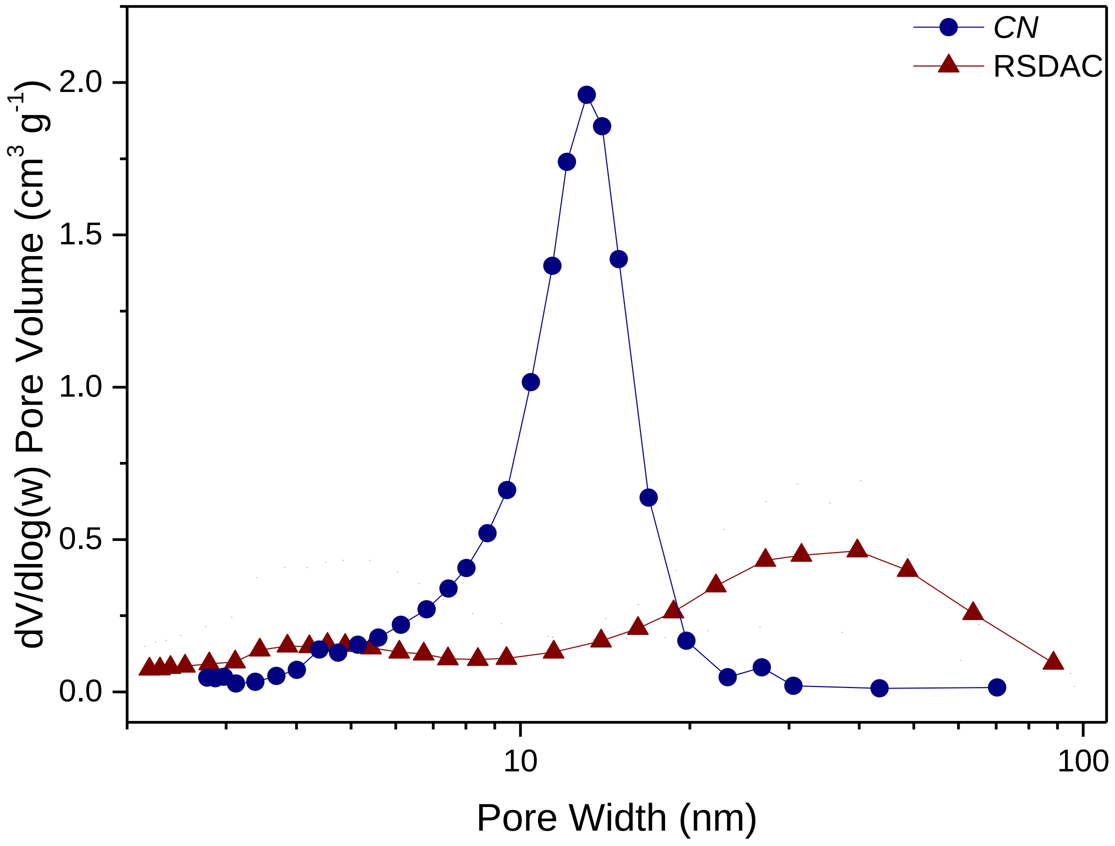

Figure S1 – Pore size distribution of Cladophora nanocellulose and RSDAC beads

Control experiments for the retention of blood activation markers.

Control experiments were performed to investigate if blood activation markers were retained by the materials. The cellulosic materials were incubated with standard solutions of the activation markers in the same conditions as in the loop experiments. The studied materials (5 mg mL^{-1}) were incubated with a standard solution of TAT (14.6 μg L^{-1}) during 1 h, under rotation at 37 ºC. The dispersion was centrifuged at 1000g for 15 min and the supernatants collected and analysed by ELISA. The TAT values obtained before (control) and after incubation with the materials were compared to each other to determine if the samples significantly retain TAT.

For C3a and sC5b-9 the cellulosic samples were incubated with Zymosan activated serum (0.7 μg L^{-1} C3a and 7.8 AU mL^{-1} sC5b-9), following the same procedure as described for TAT.

Table S1. Levels of TAT, C3a and sC5b-9 after standard solution incubations with the studied materials.

	**Control**	**Cladophora nanocellulose**	**RSDAC**
**TAT (μg L^{-1})**	19.7±2.9	12.1±2.9*	18.2±2.9
**C3a (μg L^{-1})**	0.7±0.1	ND	ND
**sC5b-9 (AU mL^{-1})**	7.8±1.1	5.5±1.1*	6.4±1.1*

* Statistically significant differences between control (before incubation) and after incubation with the materials ($p < 0.05$)

ND= non-detectable
